# Absence of the Periodontal Space in Impacted Third Molars: A Cross-Sectional Study Using Cone Beam Computed Tomography

**DOI:** 10.4317/jced.63920

**Published:** 2026-03-30

**Authors:** Cinthia Christhie Sancho Davila-Moreno, Mary Isolina Mendez-Nava, Jhoana Mercedes Llaguno-Rubio, Gustavo Adolf Fiori-Chincaro, Luis Ernesto Arriola-Guillén

**Affiliations:** 1 Division of Oral and Maxillofacial Radiology, Faculty of Dentistry, Centro Universitário do Norte de São Paulo (UNORTE), São Paulo, Brazil; 2 Division of Oral Radiology, Instituto Latinoamericano de Altos Estudios en Estomatología (ILAE), Lima, Peru; 3 Division of Orthodontics, Faculty of Dentistry, Universidad Científica del Sur, Lima, Peru

## Abstract

**Background:**

This study aimed to determine the frequency of tomographic signs indicating the absence of the periodontal space (APS) in impacted third molars using cone-beam computed tomography (CBCT) and to assess its association with demographic and anatomical variables, as well as the presence of replacement resorption (RR).

**Material and Methods:**

A cross-sectional and retrospective observational study was conducted using 258 randomly selected tomographic volumes from a radiological center in Lima, Peru, in 2025. The study evaluated 457 impacted upper and lower third molars. Two trained and calibrated evaluators analyzed the impaction of the third molars using multiplanar CBCT reconstructions with a voxel size of 0.2 mm. The variables assessed included age, sex, dental arch, third molar position, affected root zone, angulation of the third molar (APS), and root resorption (RR). Chi-square tests and logistic regression analyses were performed, with p &lt; 0.05 considered statistically significant.

**Results:**

221 cases (48.35%) exhibited an absence of periodontal ligament spaces. The absence of the periodontal space was significantly associated with third molar position, being more frequent in horizontal (27.1%) and distoangular (23.1%) positions (p = 0.003). The apical third was the most commonly affected root zone (43.0%) (p &lt; 0.001). Replacement resorption (RR) was observed exclusively in molars without a detectable periodontal space (3.6%) (p = 0.003). Age was identified as the only significant predictor; with each additional year, the risk of APS increased by a factor of 1.07 (p &lt; 0.001).

**Conclusions:**

The absence of the periodontal ligament space in third molars is highly prevalent, especially in older patients, particularly those with horizontal or distoangular positions and when the apical region is involved.

## Introduction

The periodontal ligament, along with the radiolucent space visible in radiographic imaging, is an important structural element that separates the root cementum from the alveolar bone. This separation allows for fiber attachment, vascularization, and physiological tooth mobility. However, the loss or radiographic obliteration of this periradicular radiolucent line indicates pathological issues such as dentoalveolar ankylosis or replacement resorption (RR), which can compromise the function and prognosis of the affected tooth (1 , 2). In third molars, changes in the periodontal space are especially significant. These teeth often face issues such as retention or impaction, are frequently involved in complex surgical procedures, and the condition of their roots directly influences surgical planning and associated risks (3 - 5). Traditionally, two-dimensional radiographic techniques, such as periapical and panoramic radiographs, have been used to evaluate periodontal ligament conditions. However, these methods have significant limitations for assessing fine three-dimensional structures. Problems such as anatomical superimposition and low sensitivity in detecting partial or early involvement of the periodontal space severely limit their effectiveness (6 , 7). In contrast, cone-beam computed tomography (CBCT) offers multiplanar and volumetric reconstructions with sufficient resolution when optimal protocols are used, allowing for a more accurate visualization of the continuity or loss of the periodontal space, as well as the detection of signs of ankylosis or early resorption (4 , 5 , 8 , 9). Recent studies using CBCT have estimated the prevalence of ankylosis with replacement resorption in impacted teeth at 2-6%. These studies have also found that the likelihood of replacement resorption increases with age and varies according to tooth type, with third molars exhibiting some of the lowest prevalence rates (10 - 12). Despite these advancements, there remains a lack of specific evidence on the prevalence of the absence of the periodontal space in third molars assessed via CBCT, particularly within Latin American populations (14 - 16). Much of the existing research has focused on damage to adjacent teeth, methodological simulations, or other tooth types, leaving a gap in the direct estimation of this condition in third molars. Therefore, it is crucial to generate recent local data that quantifies the frequency of this condition and examines its association with demographic and topographic variables. This information will guide clinical decision-making, diagnostic protocols, and referral criteria for the use of cone-beam computed tomography in these teeth. In this context, the present study aims to determine the frequency of tomographic signs indicating the absence of the periodontal space in upper and lower third molars and to analyze its associations with age, sex, the maxilla versus mandible, and root zone.

## Materials and Methods

**Figure 1:**
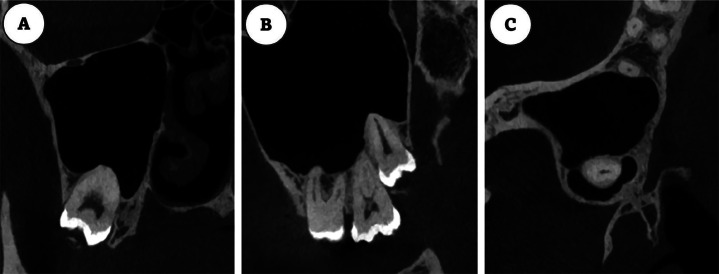
Visualization of three tomographic slices of an impacted upper third molar shows the absence of the lower periodontal ligament in certain segments of its perimeter: A. Coronal section, B. Sagittal section, C. Axial section.

**Figure 2:**
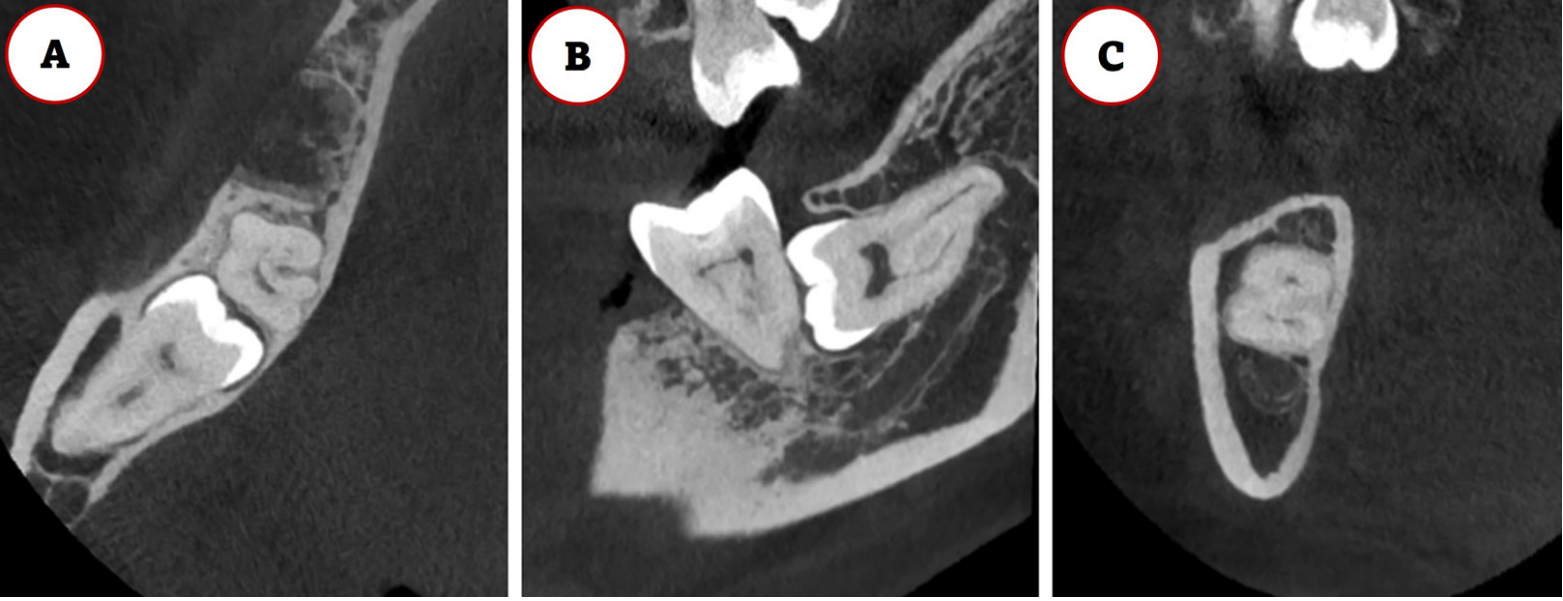
Visualization of three tomographic slices of an impacted lower third molar shows the absence of the lower periodontal ligament in certain segments of its perimeter: A. Coronal section, B. Sagittal section, C. Axial section.

- Study Design and Ethical Approval The present study was a cross-sectional, retrospective analysis approved by the Institutional Review Board of the Centro Universitário do Norte de São Paulo (UNORTE) in São Paulo, Brazil (protocol number 011-2026). It adhered to all ethical standards, ensuring the confidentiality of patient data in accordance with the Declaration of Helsinki to prevent any potential identification of individuals. - Sample Size The study population consisted of 3,718 tomographic volumes obtained from January to June 2025 at a private radiological center in Lima, Peru. A total of 258 medium-field-of-view tomographic volumes were selected using simple random sampling. The study evaluated 457 impacted upper and lower third molars. The sample size was calculated based on an expected prevalence of 24%, a 95% confidence level, and a 5% margin of error. https://www.fisterra.com/formacion/metodologia-investigacion/determinacion-tamano-muestral/#sec4 - Selection Criteria Inclusion criteria for the tomographic volumes comprised patients older than 20 years, with recorded age and sex, and images of adequate quality. Volumes that exhibited reduced image quality, artifacts, low diagnostic quality, or the presence of pathologies that hindered the evaluation of the third molar were excluded. - Image Acquisition Image acquisition was conducted using a Finnish-made PLANMECA unit, specifically the ProMax 3D MAX model, with acquisition parameters ranging from 1 to 12 mA and 60 to 120 kVp. The images were analyzed using Planmeca Romexis® software, version 6, with a voxel size of 0.2 mm. Evaluations were performed on axial, coronal, and sagittal sections, with the root divided into cervical, middle, and apical thirds. - Training and Calibration A training and outcome assessment plan was implemented through a pilot test involving the investigators. Two radiologists conducted all measurements, with a specialist in Oral and Maxillofacial Radiology, with over 10 years of experience, serving as the gold standard. Inter- and intra-examiner agreement tests were conducted, and calibration was considered acceptable when the Kappa coefficient exceeded 0.8. For this purpose, 10% of the sample was used and was later included in the final evaluation. - Variable Measurements The following variables were recorded: age, sex (male or female), dental arch (upper or lower), and the position of the third molar according to Winter's classification (vertical, mesiangular, distoangular, horizontal, transverse, or inverted). Additionally, the affected root zone was noted (cervical, middle, apical, two zones, or all zones), as well as the occurrence of replacement resorption (present or absent) and the presence or absence of the periodontal space. This last assessment involved analyzing three tomographic slices of the impacted third molar to identify any absence of the lower periodontal ligament in specific segments of its perimeter (Figs. 1,2).

1

2

- Statistical Analysis Statistical analysis was performed using SPSS Statistics (version 24.0; IBM, Armonk, NY). Descriptive analysis was conducted using frequencies and percentages. Chi-square tests were employed to evaluate bivariate associations, and logistic regression was applied to identify predictors of APS. A p-value of less than 0.05 was considered statistically significant.

## Results

Out of 457 evaluated upper and lower third molars, 221 cases (48.35%) exhibited an absence of periodontal ligament spaces. The initial characteristics of the sample are detailed in Table 1.

Table 1

The relationship between the presence of periodontal spaces and the impaction position of third molars is evaluated in Table 2.

Table 2

It was found that the absence of periodontal spaces was more frequent in the horizontal (27.1%) and distoangular (23.1%) impaction positions. Conversely, periodontal spaces were more commonly observed in the mesioangular (32.2%) and horizontal (28.4%) positions (p = 0.003). Table 3 illustrates the relationship between the presence of periodontal spaces and the impaction zone of third molars.

Table 3

It shows that periodontal spaces are more often absent in the apical third (43%) than when present, and most cases with spaces did not show impacted third molars (p&lt;0.001). Table 4 shows the relationship between the presence of periodontal spaces and the occurrence of replacement root resorption.

Table 4

Among cases without periodontal spaces, 3.6% exhibited signs of replacement root resorption. However, none of the cases with periodontal spaces showed any evidence of this type of resorption (p = 0.003). Table 5 assesses how various predictor variables-specifically sex, age, arch, and third molar position-affect the visibility of periodontal ligament spaces in third molars.

Table 5

The analysis reveals that only age significantly influences this outcome. For each additional year of age, the risk of not having periodontal ligament spaces increases 1.07 times (p &lt; 0.001)

## Discussion

The present study examined the frequency and factors associated with the absence of the periodontal space (APS) in impacted third molars using cone-beam computed tomography (CBCT). This research provides three-dimensional evidence in an adult Peruvian population. The findings are clinically significant because the absence of the periodontal space is a radiographic indicator of dentoalveolar ankylosis and potential replacement resorption (RR), both of which can impact the complexity of surgical procedures and influence clinical decision-making (1 , 2 , 14). The results showed that APS was significantly associated with third molar position, with a higher frequency in horizontal and distoangular positions. This observation is consistent with previous studies reporting a higher likelihood of close contact between the root surface and the alveolar bone in these positions, which favors the progressive loss of the periodontal ligament due to continuous bone remodeling (3 , 10 , 12 , 15). The literature indicates that horizontally impacted third molars are subjected to constant mechanical forces and prolonged lack of eruptive function, factors that may induce localized ankylosis phenomena (16 , 17). In contrast, when the periodontal space was present, the mesioangular position predominated (32.2%), with statistically significant results (p = 0.003). These percentages suggest that horizontal and distoangular positions, characterized by greater root-surface contact with the alveolar bone, may favor progressive loss of the periodontal ligament (3 , 10 , 12 , 15). This behavior is consistent with previous reports of a higher frequency of ankylosis and increased surgical complexity in third molars with these angulations, particularly when impaction persists over prolonged periods (4 , 11). In this context, the force vector and mechanical pressure exerted by the third molar in these positions on adjacent osseous structures may constitute determining factors in the loss of the periodontal space (16 , 17). Regarding the affected root zone, the results showed that APS was predominantly observed in the apical third, accounting for 43.0% of cases without periodontal space, followed by involvement of two root zones (26.7%) and, finally, single-root-zone involvement (23.5%). This pattern is consistent with previous three-dimensional reports indicating that the apical region is particularly susceptible to degenerative changes due to its relatively lower blood supply and its close relationship with the adjacent trabecular bone (8 , 18 , 19). Moreover, assessment of this region on two-dimensional radiographs is limited by anatomical superimposition, underscoring the diagnostic value of CBCT for early detection of periodontal ligament alterations (6 , 7 , 20). A clinically relevant finding was the relationship between the absence of periodontal space and replacement root resorption (RR). In the present study, 3.6% of third molars without a periodontal space showed tomographic signs of RR, whereas no cases (0%) with a visible periodontal space exhibited this type of resorption. This difference was statistically significant (p = 0.003), supporting the pathophysiological concept that loss of the periodontal ligament constitutes an indispensable prerequisite for bone-root fusion and subsequent replacement resorption (2 , 10 , 14 , 21). Although the absolute frequency of RR was low (1.8%), this association is consistent with previous studies reporting low but clinically relevant rates of RR in impacted teeth evaluated using CBCT (9 , 22). The study identified age as the only significant factor associated with APS. Specifically, for each additional year of age, the probability increased by 1.07 times (p &lt; 0.001). This finding is consistent with longitudinal and cross-sectional studies reporting a progressive increase in ankylosis and replacement resorption in retained teeth with advancing patient age (10 , 17 , 23). It supports the notion that prolonged dental retention is associated with progressive degenerative changes in the periodontal ligament, driven by reduced cellular activity, narrowing of the periodontal space, and a predominance of bone remodeling processes (18 , 23). In contrast, variables such as sex, dental arch, and third molar position did not show a significant association in the adjusted model, suggesting that age has a more determinant influence than anatomical characteristics. This finding is consistent with recent studies that have identified age as the main risk factor for periodontal space loss and the development of ankylosis in impacted teeth (10 , 23). This study has some limitations. Its cross-sectional design prevents us from making causal inferences, and the evaluation of the periodontal space is dependent on the quality of the images. However, the use of CBCT with standardized protocols and thorough three-dimensional analysis enhances the validity of the findings. Future longitudinal studies will help assess the absence of periodontal space and its direct relationship with the development of ankylosis and replacement resorption.

## Conclusions

The absence of periodontal ligament space in third molars is quite common. This phenomenon is especially noticeable in the apical third of the tooth, particularly in those that are horizontally or distoangulary positioned and among older individuals. It should be regarded as a significant warning sign on tomographic images. Detecting this condition on CBCT may indicate a higher likelihood of ankylosis and/or replacement resorption, which can complicate surgical procedures and necessitate adjustments to treatment planning.

## Figures and Tables

**Table 1:** Table Initial characteristics of the sample (n=258).

	Age			
Sex	n	Mean	SD	p
Female	141	43.57	16.30	0.836
Male	117	44.00	16.52	

T-test

**Table 2:** Table Relationship between the presence of periodontal space and the position of third molar impaction.

Periodontal spaces		Position of the impacted third molar						
		Vertical	Mesioangular	Distoangular	Horizontal	Transversal	Invert	Total
Absent	n	41	40	51	60	27	2	221
	%	18.6	18.1	23.1	27.1	12.2	0.9	100.0
Present	n	39	76	40	67	13	1	236
	%	16.5	32.2	16.9	28.4	5.5	0.4	100.0
Total	n	80	116	91	127	40	3	457
	%	17.5	25.4	19.9	27.8	8.8	0.7	100.0

P=0.030Chi Square Test

**Table 3:** Table Association between the presence of periodontal spaces and the impaction zone of the third molars.

Periodontal spaces		Impaction zone of the impacted third molar						
		Absent	Cervical	Medium	Apical	Two zones	All zones	Total
Absent	n	3	3	9	95	59	52	221
	%	1.4	1.4	4.1	43.0	26.7	23.5	100.0
Present	n	228	0	1	4	1	2	236
	%	96.6	0.0	0.4	1.7	0.4	0.8	100.0
Total	n	231	3	10	99	60	54	457
	%	50.5	0.7	2.2	21.7	13.1	11.8	100.0

P<0.001

**Table 4:** Table Association between the presence of periodontal spaces and the presence of replacement root resorption.

Periodontal spaces		Root resorption due to replacement		
		Absent	Present	Total
Absent	n	213	8	221
	%	96.4	3.6	100.0
Present	n	236	0	236
	%	100.0	0.0	100.0
Total	n	449	8	457
	%	98.2	1.8	100.0

P=0.003Chi-Square Test

**Table 5:** Table Influence of predictor variables on the appearance of spaces in the periodontal ligament of impacted third molars.

Predictor variables	p	Exp(B)	C.I. to 95% for B	
			Lower	Upper
Female	___	___	___	___
Male	0.841	0.94	0.54	1.66
Age	<0.001*	1.07	1.05	1.09
Upper arch	___	___	___	___
Lower arch	0.180	0.68	0.39	1.19
Third molar position	0.136	1.18	0.95	1.46

* Significant

## Data Availability

The data supporting the findings of this study are available from the corresponding author upon reasonable request.
